# Dynamic coherence windows: a multi-scale structural coherence measurement framework for AI systems

**DOI:** 10.3389/frai.2026.1836120

**Published:** 2026-05-21

**Authors:** Christian St-Louis

**Affiliations:** Systèmes de Cohérence Coherix Inc., Mirabel, QC, Canada

**Keywords:** coherence measurement, controlled evaluation, LLM evaluation, multi-scale analysis, structural coherence, viability theory

## Abstract

Assessing the structural integrity of AI system outputs is typically based on surface-level metrics such as fluency or local coherence. This paper proposes an alternative perspective: measuring structural coherence as a multi-scale property of system outputs. We introduce the Dynamic Coherence Window (DCW) framework, which models structural viability as bounded behavior within coherence thresholds derived from the system’s internal organization. The framework defines a set of structural defect variables capturing distinct modes of degradation, along with two key state variables—the coherence margin m and stability parameter *Λ*—that jointly characterize system state. As a conceptual extension, we introduce the Cognitive Immune System (CIS), a theoretical evaluation mechanism that assesses the structural impact of new information prior to integration. The CIS defines four decision pathways (accept, reject, quarantine, reframe), including a human-mediated mechanism for integrating structurally incompatible inputs. We present CMCI v8.1.4, an implementation of the framework, and evaluate it through large-scale simulation (*N* = 10,000), targeted text analysis (*N* = 21), a controlled human-vs-LLM benchmark (*N* = 60), and cross-benchmark evaluation (*N* = 96). Results are consistent with the interpretation that the framework measures a structural dimension of coherence distinct from surface-level metrics under controlled conditions. These findings provide preliminary empirical support for the hypothesis that structural coherence constitutes a measurable and distinct dimension of system output quality. The present work introduces and evaluates a structural coherence measurement framework under controlled conditions. Validation in real-world deployment settings remains an open direction for future research.

## 1 Introduction

The rapid deployment of large language models and AI systems has highlighted the need for methods that assess output quality beyond surface-level fluency. Existing evaluation approaches largely focus on output acceptability, either through training-time optimization (e.g., reinforcement learning from human feedback) or inference-time filtering and post-processing. In both cases, quality is primarily treated as a property of individual outputs.

This work adopts a complementary perspective. Rather than focusing solely on output acceptability, we investigate whether structural coherence can be defined, measured, and tracked as a continuous property of system outputs. This distinction is important: outputs may appear locally acceptable while underlying structural properties vary across conditions. Measuring structural coherence may therefore provide additional diagnostic information not captured by output-level evaluation.

Importantly, the proposed framework is not intended to replace existing evaluation approaches, but to introduce a complementary measurement dimension that operates alongside standard methods.

To formalize this perspective, we introduce the Dynamic Coherence Window (DCW) framework. The central idea is that system outputs can be characterized relative to a bounded region of structural coherence, defined through thresholded constraints on internal relationships. When these constraints are maintained, outputs remain structurally consistent under the defined conditions; deviations from these bounds correspond to measurable structural changes.

As a conceptual extension, we introduce the Cognitive Immune System (CIS), a theoretical construct designed to explore how structural coherence might be evaluated prior to integrating new information. The CIS defines four decision pathways (accept, reject, quarantine, reframe), which serve as an abstract model for reasoning about structural compatibility. This component is not implemented as an operational system, but is included to illustrate potential extensions of the framework.

The contributions of this work are as follows:

1. A formal framework for defining and measuring structural coherence as a multi-scale property of system outputs;
2. A characterization of structural regimes under controlled conditions;
3. A conceptual extension (CIS) illustrating how structural evaluation could be applied to information integration;
4. Empirical evaluation through simulation and controlled text-based experiments, providing preliminary evidence that the proposed framework captures structural properties distinct from conventional metrics;
5. A proof-of-concept benchmark comparing human and LLM outputs, demonstrating that the framework can distinguish structural coherence from surface-level fluency under the tested conditions.

## 2 Related work

### 2.1 Output-level evaluation and alignment

The dominant paradigm in AI system evaluation focuses on aligning model outputs with human preferences. Reinforcement learning from human feedback (RLHF) and related approaches optimize model behavior to produce responses that are judged acceptable by human evaluators (Ouyang et al., 2022; Bai et al., 2022a, 2022b). These methods have been effective in reducing undesirable outputs. Beyond direct preference modeling, the broader AI safety literature has identified a range of structural concerns affecting model behavior, including specification gaming and reward hacking (Amodei et al., 2016), unresolved problems in robustness, monitoring, and systemic safety (Hendrycks et al., 2022), and the broader alignment problem from a deep learning perspective (Ngo et al., 2023). Studies on detoxification have also highlighted that surface-level interventions can lead to coverage trade-offs (Welbl et al., 2021).

However, these approaches primarily evaluate quality at the level of individual responses and do not explicitly characterize structural properties across outputs. Recent work has highlighted that model behavior may vary under different conditions, including prompt variation and distributional shifts (Qi et al., 2024; Confident AI, 2025).

In this context, the Dynamic Coherence Window (DCW) framework is intended as a complementary perspective, focusing on the characterization of structural coherence as a measurable property under controlled conditions, rather than on output-level evaluation alone.

### 2.2 Guardrails and output-level constraints

The deployment of large language models has led to the development of guardrail systems designed to constrain model behavior at inference time. Frameworks such as NeMo Guardrails (Rebedea et al., 2023) and Guardrails AI implement rule-based filtering and output validation, and recent surveys have proposed systematic approaches to construct guardrails for LLMs across diverse contexts (Dong et al., 2024), while more recent approaches incorporate learned safety classifiers.

These methods primarily operate at the semantic level, focusing on evaluating and constraining generated outputs. While effective in mitigating undesirable responses, they do not explicitly characterize structural properties of outputs across scales.

The present framework is not designed as a guardrail or runtime system. Instead, it introduces a complementary measurement perspective, focusing on the characterization of structural coherence as a measurable property under controlled evaluation conditions.

### 2.3 Viability theory

Viability theory provides a mathematical framework for analyzing systems that must remain within constraint sets to persist over time (Aubin, 1991; Aubin et al., 2011). A central concept is the viability kernel, defined as the set of states from which the system can evolve without violating its constraints.

The present work draws inspiration from this framework by treating coherence as a bounded property defined by constraint satisfaction. Rather than assuming fixed thresholds, the framework explores how such constraints may be operationalized as measurable quantities under controlled conditions.

### 2.4 Coherence in cognitive science

The concept of coherence has been studied across multiple disciplines. In linguistics, cohesion contributes to textual unity (Halliday and Hasan, 1976). In cognitive neuroscience, coherence has been associated with large-scale coordination (Varela et al., 2001). These perspectives motivate the treatment of coherence as a multi-scale property. This multi-scale approach is consistent with broader perspectives on complexity (Morin, 1992) and unified theoretical frameworks linking multi-scale interactions to system viability (Friston, 2010). The notion of structural robustness across scales has also been emphasized in biological systems, where the maintenance of function despite perturbations reflects underlying coupling between subsystems (Kitano, 2004).

The present framework adopts this perspective by defining structural coherence as an emergent property arising from relationships between constraints across scales, without assuming a specific underlying cognitive mechanism.

### 2.5 Critical transitions and early warning

Research on critical transitions in complex systems has shown that abrupt changes in behavior may be preceded by identifiable signals (Scheffer et al., 2009). Such indicators characterize system state relative to stability boundaries.

In this work, the coherence margin m and stability parameter *Λ* are introduced as quantities that characterize system state relative to defined structural constraints. These quantities are not direct analogues of classical early-warning signals but provide a means of describing system behavior under controlled experimental conditions.

### 2.6 Immune-inspired models

Biological immune systems have inspired computational models for anomaly detection, particularly in artificial immune systems (AIS) (Dasgupta, 1999; De Castro and Timmis, 2002). These approaches typically rely on detecting deviations from expected patterns.

The Cognitive Immune System (CIS) proposed in this work is a conceptual extension used to illustrate how structural coherence might be evaluated prior to integrating new information. It is not implemented as an operational system but serves as a theoretical construct for reasoning about structural compatibility.

## 3 The dynamic coherence window framework

### 3.1 Defect variables

We represent system state using a set of five defect variables D = (d₁, d₂, d₃, d₄, d₅), each corresponding to a distinct mode of structural degradation. These variables are defined as abstract structural dimensions characterizing different types of coherence loss.

The five defect variables are defined as follows:

- d₁ (fragmentation): loss of continuity in reasoning structure.
- d₂ (misalignment): internal inconsistency across components.
- d₃ (instability): irregular or recursive variation in outputs.
- d₄ (opacity): reduced interpretability of output structure.
- d₅ (rigidity): reduced adaptability across response patterns.

Each defect variable satisfies dᵢ ≥ 0 and represents deviation from an ideal coherent state along a specific dimension.

These variables are not directly observed. Their operational estimation within the CMCI framework is defined separately in Section 4.5, where abstract defect dimensions are approximated using measurable components derived from system outputs under controlled conditions.

### 3.2 Thresholds

The framework defines a set of thresholds δᵢ associated with each defect variable. These thresholds are treated as parameters specifying acceptable ranges under the evaluation conditions.

In contrast to fixed-threshold approaches, the framework allows these thresholds to vary depending on system configuration and experimental setup. In the present study, thresholds are treated as calibrated values determined prior to evaluation.

### 3.3 Coherence window and margin

The coherence window W is defined as the set of states for which all defect variables remain within their thresholds:

$W=\{D\;|\forall i:d_{i}<\delta_{i}\}$

The coherence margin m is defined as:

$m=\min_{i}\;(\delta_{i}-d_{i})$

Interpretation:

- m > 0 indicates that the system remains within the coherence window
- m = 0 indicates boundary conditions
- m < 0 indicates violation of coherence constraints

For empirical evaluation, a reduced scalar approximation of the margin is used:

m=R−F

where R represents adaptive repair capacity and F represents accumulated destabilization. This approximation preserves the sign structure of the full margin while enabling tractable computation. All reported empirical results are based on this reduced representation.

### 3.4 Stability parameter

The stability parameter *Λ* is introduced as a scalar quantity characterizing the balance between stabilizing and destabilizing effects:

Λ=(γ·n_eff)/A_eff

where γ represents effective coupling strength, n_eff the number of active constraints, and A_eff the effective degradation rate.

This parameter is used as a descriptive quantity rather than a fully derived dynamical invariant.

### 3.5 Regime characterization

System behavior can be qualitatively described in terms of the coherence margin m and stability parameter *Λ*:

- Stable: m > 0 and Λ > 1.
- Near-critical: m → 0.
- Incoherent: m < 0.
- Recovery: m increasing toward positive values.

**Figure 1:**
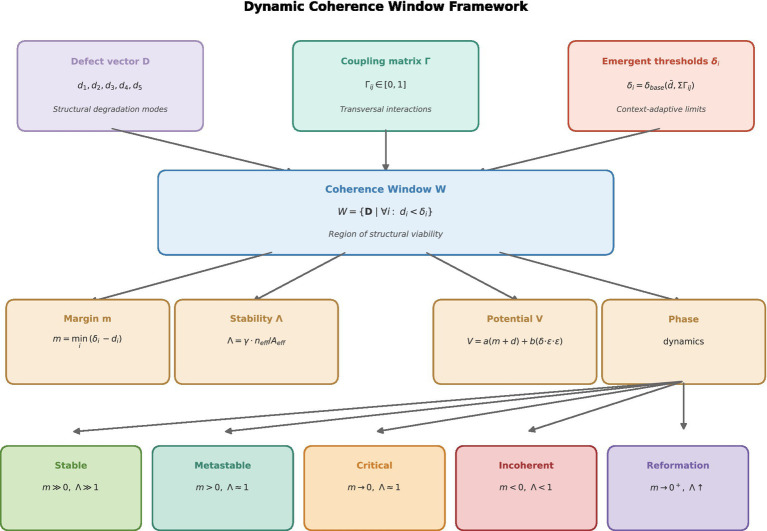
Conceptual framework. The dynamic coherence window framework is presented as a structural viability system in which the defect vector D, the coupling matrix *Γ*, and emergent thresholds δ_i_ together define a bounded region of operation—the coherence window W. Within this region, system states remain viable, while proximity to the boundary is captured by the coherence margin *m*. The stability parameter *Λ* modulates resilience through coupling intensity and effective constraint activation. Together, these elements define a phase structure linking structural degradation to qualitative system behavior and provide the theoretical foundation for coherence-based structural evaluation under the proposed framework.

**Figure 2:**
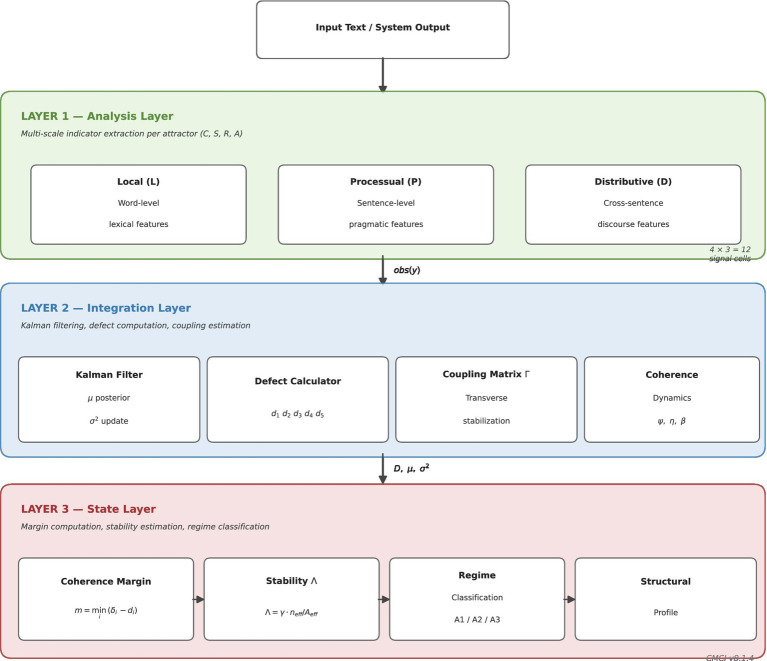
System architecture. Three-layer architecture of CMCI v8.1.4 comprising (i) an analysis layer for extracting multi-scale indicators, (ii) an integration layer for defect aggregation and coupling estimation, and (iii) a state layer for margin computation, stability estimation, and regime classification. This architecture operationalizes the CMCI framework as an analysis pipeline from raw system outputs to structural viability indicators, enabling structural coherence evaluation under controlled conditions.

These regimes provide an interpretive framework for describing system behavior under the evaluated conditions, without assuming a specific underlying dynamic model (see Figures 1, 2).

## 4 Methods

The CMCI formulation can be interpreted as a reduced projection of the DCW framework, in which the high-dimensional defect representation is mapped into a lower-dimensional space for empirical evaluation. CMCI provides an operational approximation of the framework, enabling tractable computation under controlled conditions.

### 4.1 Coherence margin computation

The CMCI framework evaluates structural coherence through a multi-stage pipeline. For each generated text or system output, five coherence components are computed:

1. Contradiction Density (E): logical inconsistencies within the text
2. Contextual Drift (S): deviation from the initial semantic context
3. Internal Tension (C): competing semantic signals
4. Cross-scale Misalignment (T): incoherence across structural levels
5. Recursive Instability (F): amplification of inconsistencies across reasoning steps

All reported values correspond to deterministic runs with fixed random seeds unless otherwise specified.

These components are aggregated into a global coherence representation. The coherence margin m is computed as:

m=R−F

where R represents adaptive coherence capacity and F represents accumulated destabilization.

This formulation preserves the sign structure of the coherence margin and provides a tractable scalar approximation for empirical evaluation under the tested conditions.

### 4.2 Regime classification

System states are classified into three regimes based on the coherence margin:

- A1 — Stable: m > 0
- A2 — Critical: m ≈ 0
- A3 — Unstable: m < 0

These regimes provide a descriptive categorization of system behavior under the evaluation conditions.

### 4.3 Simulation protocol

To evaluate CMCI behavior at scale, we generate *N* = 10,000 samples through uniform sampling over the space [0,1]^2^. For each sample, the coherence margin is computed via the CMCI pipeline, and a regime is assigned based on thresholding.

Model parameters are calibrated to ensure: (i) separation between regimes; (ii) presence of a non-trivial critical region; and (iii) absence of degenerate behavior. Random seeds are fixed to ensure reproducibility.

### 4.4 CMCI pipeline (pseudocode)

The computational pipeline proceeds as follows:

(1) Segment the input into analysis units.
(2) Compute embeddings for each segment.
(3) Evaluate coherence components (E, S, C, T, F)
(4) Aggregate components into a global representation.
(5) Compute the coherence margin m = R – F.
(6) Assign regime (A1, A2, A3) based on margin thresholds.
(7) Optionally perform exploratory analysis, including tracking of m across steps.

This pipeline defines the operational procedure used in all empirical evaluations reported in this work.

### 4.5 Operational mapping of defect dimensions

To connect the theoretical defect variables defined in Section 3.1 with the CMCI implementation, we provide an operational mapping between the abstract dimensions (d₁–d₅) and the measurable components computed by the CMCI pipeline. For clarity, the notation d₁–d₅ consistently refers to the abstract defect variables defined in Section 3.1, while E, S, C, T, F denote the measurable proxy components used for their operational approximation within the CMCI framework.

- d₁ (fragmentation) is associated with Contextual Drift (S) and Cross-scale Misalignment (T).
- d₂ (misalignment) is associated with Internal Tension (C) and Cross-scale Misalignment (T).
- d₃ (instability) is associated with Recursive Instability (F).
- d₄ (opacity) is associated with Contradiction Density (E).
- d₅ (rigidity) is associated with reduced variability across coherence components.

This mapping provides an operational bridge between the theoretical framework and the empirical measurements used in this study. It defines how abstract defect variables are approximated using measurable proxy components derived from system outputs under controlled experimental conditions, rather than being directly observed.

## 5 Cognitive immune system (conceptual extension)

The Cognitive Immune System (CIS) is introduced as a conceptual extension of the DCW framework. In the present study, it is not treated as a validated operational system, but as a theoretical construct illustrating how structural coherence assessment could be applied to the evaluation of new information prior to integration.

### 5.1 Conceptual architecture

The CIS can be described as a conceptual evaluation structure in which candidate inputs are assessed before integration. It is represented through a set of functional roles:

1. Detection: identification of incoming inputs for evaluation
2. Evaluation: analysis of structural coherence under different scenarios
3. Comparison: assessment of compatibility with the current system state
4. Decision: selection of an appropriate outcome based on coherence criteria
5. Memory: recording of prior evaluations for reference
6. Logging: traceability of evaluation steps and outcomes

These elements are not implemented as a standalone system but provide an abstract framework for reasoning about coherence-based evaluation.

### 5.2 Decision pathways

Within this conceptual framework, four possible outcomes are defined:

- Accept: the input is compatible with structural coherence
- Reject: the input is incompatible under the defined criteria
- Quarantine: the impact is uncertain and requires further evaluation
- Reframe: the input may become compatible under additional contextual interpretation.

These pathways define a conceptual decision space for analyzing how structural constraints may influence information integration.

### 5.3 Human coherence bridging

The reframe pathway introduces the concept of human coherence bridging. In this scenario, additional contextual input from a human operator may enable reinterpretation of an otherwise incompatible input.

This mechanism is presented as a conceptual illustration rather than an implemented process. It highlights how contextual framing may influence structural compatibility without modifying the original input.

This section is intended to provide an illustrative extension of the measurement framework and does not constitute an operational component of the CMCI system in the present study.

## 6 Experiments

See Table 1.

**Table 1:** Summary of experimental evaluations.

Section	Experiment	Description	Sample size (*N*)	Type
6.1–6.5	Simulation study	Synthetic risk-space sampling and regime analysis	10,000	Controlled
6.6	Multiscale dynamic simulation	Exploratory multi-domain temporal simulation	—	Exploratory
6.7	Human vs. LLM benchmark	Structured comparison across quality tiers	60	Controlled
6.8	Exploratory qualitative assessment	Informal qualitative evaluation scenarios	—	Exploratory
6.9	Structural perturbation analysis	Controlled manipulation (intact, shuffled, etc.)	80 (20 per condition)	Controlled
6.10	Cross-benchmark evaluation	Multi-benchmark comparison (HELM, HarmBench, SOCRATES)	96	Controlled
8.1–8.3	Individual & aggregated text analysis	Small-scale structural evaluation	21	Controlled

### 6.1 Simulation setup

We conducted a large-scale simulation study to evaluate the behavior of the CMCI framework across a continuous risk space. The simulation generates *N* = 10,000 samples with risk coordinates (r_epistemic, r_stability) uniformly sampled from the domain [0, 1]^2^.

For each sample, the CMCI pipeline computes the coherence score, the margin m, and assigns a regime classification. Model parameters were calibrated to obtain an interpretable regime structure, including: (a) separation between regimes, (b) the presence of a non-degenerate critical region (A2), and (c) balanced regime occupation across samples. Random seeds are fixed to ensure reproducibility.

### 6.2 Main results: regime field

The A1 (stable) regime occupies the low-risk region where both epistemic and stability risks are limited. The A2 (critical) regime forms a transition band between stable and unstable configurations. The A3 (unstable) regime corresponds to regions where both risk components are elevated.

The observed boundaries between regimes are non-linear, consistent with interactions between epistemic and stability risk dimensions. This structure is consistent with the coherence margin formulation under the evaluated conditions.

**Figure 3:**
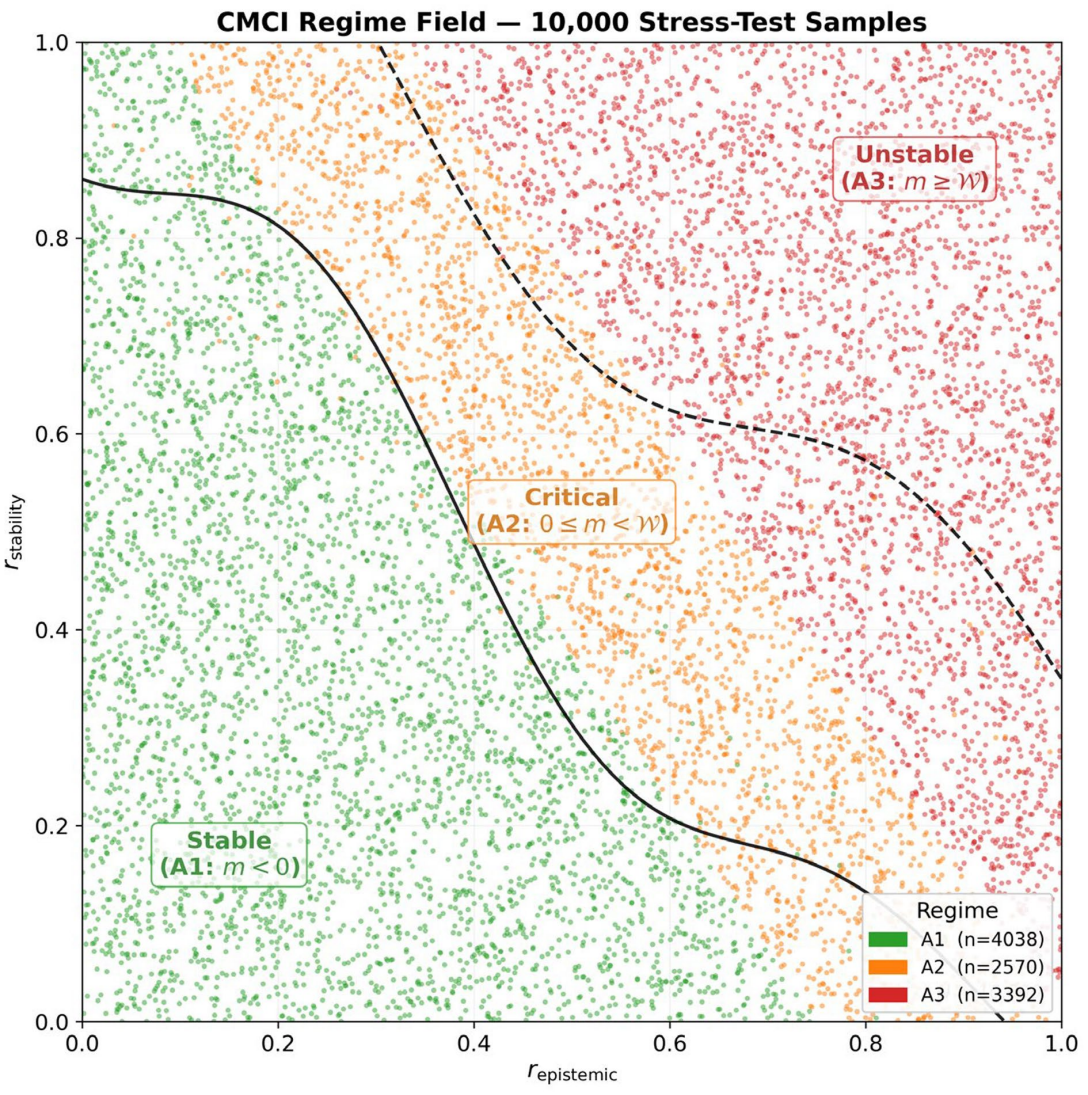
CMCI regime field. Distribution of 10,000 stress-test samples in the two-dimensional risk space, showing three non-linearly separated regimes: A1 (stable, m > 0, green), A2 (critical, |m| < *ε*, orange), and A3 (unstable, m < 0, red). The solid line indicates the m = 0 coherence boundary. The figure illustrates a structured phase space induced by the coherence margin formulation rather than by empirically inferred clustering, indicating that regime transitions arise from interaction effects between epistemic and stability risks. This is consistent with interpreting coherence as a coupled, multi-variable phenomenon under the evaluated conditions.

Minor boundary irregularities are observed near regime transitions, where the assigned regime differs from that of neighboring samples. This behavior is consistent with variability near regime boundaries and indicates that the classification is not overly rigid (see Figure 3).

### 6.3 Margin distribution

**Figure 4:**
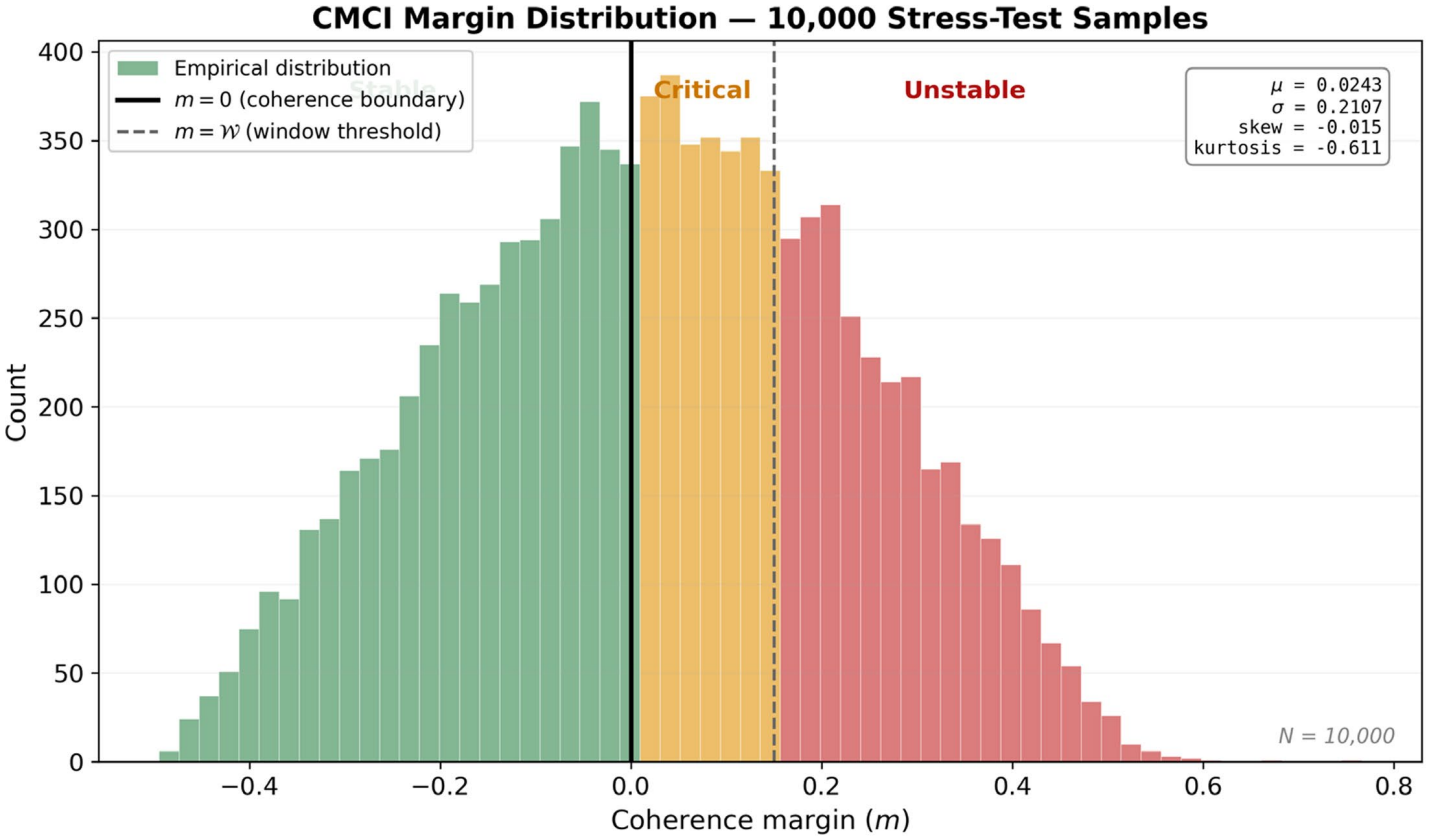
CMCI margin distribution. Distribution of the coherence margin *m* across 10,000 simulated samples, showing slight negative skew and a platykurtic shape. Colored regions correspond to regime classification: green (stable), orange (critical), and red (unstable). Statistical moments are displayed in the inset. The observed distribution is approximately symmetric and unimodal around zero, consistent with balanced sampling of positive- and negative-margin states under the evaluated conditions. These patterns are descriptive and are consistent with interpreting the coherence margin as a non-degenerate variable within the simulation setup.

The observed margin distribution, computed from the simulation outputs underlying Figure 4, exhibits the following properties: mean *μ* ≈ 0.024, standard deviation *σ* ≈ 0.211, skewness ≈ − 0.015, and kurtosis ≈ − 0.611.

The near-zero mean is consistent with the sampling procedure covering both sides of the coherence boundary. The low skewness suggests approximate symmetry of the distribution, while the negative kurtosis (platykurtic shape) indicates a relatively broad distribution without strong concentration near the mean.

Approximately 51.3% of samples fall within the coherence window (m > 0), and 48.7% fall outside (m < 0), consistent with balanced coverage of viable and non-viable regions under the sampling procedure.

### 6.4 Regime proportions

The observed regime proportions are as follows: A1 (stable): 40.4% (*n* = 4,038; 95% CI: 39.4–41.3%); A2 (critical): 25.7% (*n* = 2,570; 95% CI: 24.9–26.6%); A3 (unstable): 33.9% (*n* = 3,392; 95% CI: 33.0–34.9%).

These proportions indicate that the CMCI classification produces a non-degenerate partition of the state space under the evaluated conditions. In particular, all three regimes are represented, including an intermediate region (A2) between positive and negative margin states.

The relatively narrow 95% Wilson confidence intervals reflect the statistical precision associated with the sample size. The distribution is consistent with a balanced coverage of the sampled space rather than concentration in a single regime.

The presence of an intermediate regime indicates that states with margin values near the coherence boundary are observed within the sampled space. These observations are descriptive and remain specific to the simulation setup used in this study. These regime proportions are derived from the same 10,000-sample simulation summarized in Figures 3, 4.

### 6.5 Statistical summary

The key statistical properties of the simulation across 10,000 samples include margin distribution metrics and regime proportions. Together, these statistics provide a compact descriptive characterization of the simulated behavior and are consistent with interpreting the coherence margin as a non-degenerate variable under the evaluated conditions.

### 6.6 Multiscale and transverse dynamic simulation

To complement the static evaluation and explore potential dynamic behavior under controlled conditions, we conducted a multiscale and transversal simulation of the CMCI framework. This simulation is intended as an exploratory analysis rather than a representation of real-world system dynamics.

In this setting, the system is modeled as a multi-domain structure composed of four interacting domains (filesystem, binary, network, process), each evolving under simplified degradation and repair dynamics. For each domain, coherence is evaluated across three temporal scales: (1) Instant state L(t), representing local fluctuations; (2) Persistence P(t), computed as a moving average over a sliding window; and (3) Trajectory D(t), representing longer-term variation via slope estimation.

Transversal coherence is computed as the mean cross-domain correlation over time within this simulated environment.

#### 6.6.1 Results

The simulation reveals four distinct regimes consistent with the theoretical phase structure: Stable regime (m ≫ 0), Critical regime (m → 0), Incoherent regime (m < 0), and Reformation phase (m → 0^+^). A key observation is that resonance events co-occur with the approach to coherence boundaries in this simulated setting: cross-domain correlation increases in proximity to structural transitions; drift alignment across domains emerges as the margin approaches zero; and resonance activation is observed while m(t) is still near zero. These patterns are preliminary and have not been statistically validated.

#### 6.6.2 Interpretation

**Figure 5:**
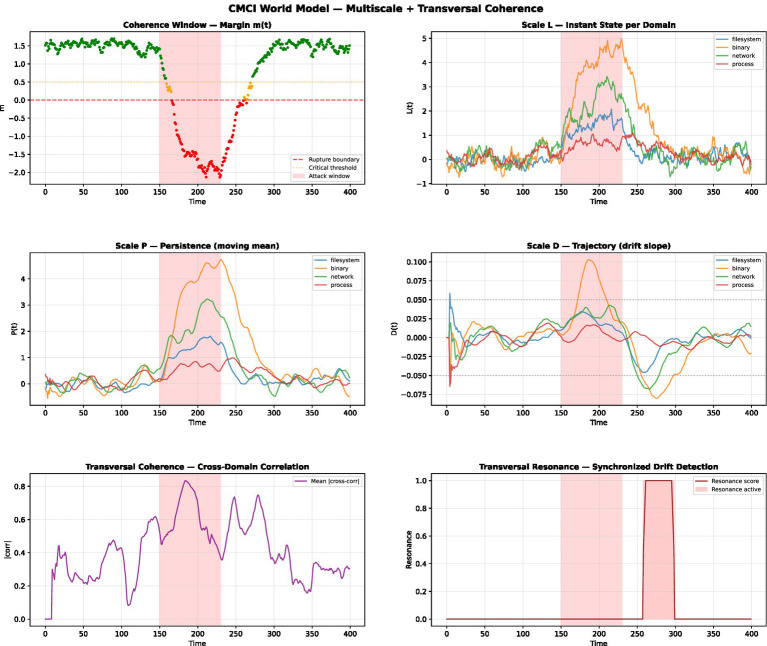
Exploratory multiscale simulation of coherence dynamics across interacting domains and temporal scales. The panels show: top-left, coherence margin m(t); top-right, instantaneous state L(t) across domains; middle-left, persistence P(t); middle-right, drift trajectory D(t); bottom-left, transversal coherence measured as cross-domain correlation; and bottom-right, a simplified indicator of synchronized variation across domains. The shaded region highlights a transition zone near the coherence boundary. The observed patterns are qualitative and intended for illustrative purposes only. This simulation is exploratory and does not constitute a validated dynamic model within the scope of the present study.

These results provide initial empirical support for the Dynamic Coherence Window framework. In particular: the coherence margin m(t) acts as a continuous indicator of system viability; multi-scale decomposition (L/P/D) reveals degradation patterns not visible at a single scale; and transversal coherence captures structural interactions that precede failure. The observation that resonance co-occurs with the approach to structural transitions in this simulated setting is consistent with the hypothesis that structural coherence signals may provide complementary information about system state, beyond what is captured by output-level evaluation alone. This interpretation remains to be validated on real-world systems (see Figure 5).

### 6.7 Human vs. LLM coherence benchmark

To evaluate whether the CMCI framework differentiates between texts of varying structural quality, we conducted a controlled benchmark comparing three categories of content: raw LLM outputs, human-revised versions of those outputs, and expert-authored texts.

### 6.8 Dataset

The benchmark corpus comprises 20 prompts, each evaluated across three quality tiers, yielding 60 samples in total. Expert texts consist of research abstracts sourced from arXiv. Raw LLM responses were generated from the same prompts, and revised texts are human-edited versions of these outputs, with improved structure and clarity while preserving the original content. All evaluations were performed using the CMCI Multiscale Analyzer.

## 7 Results

Mean CMCI coherence scores across the three tiers are as follows (mean ± standard deviation):

- Raw LLM: 0.469 (95% classified A2)
- Human-Revised: 0.593 (80% classified A1)
- Expert: 0.675 (90% classified A1)

The framework produces a consistent monotonic ordering (Expert > Human-Revised > Raw LLM) across all evaluated prompts, with no observed inversions within this sample. Of the 20 comparisons, 12 exhibit complete ordering across all three tiers, while 8 show partial overlap between adjacent score distributions while preserving overall ordering.

### 7.1 Interpretation

These results are consistent with the hypothesis that the CMCI framework captures structural differences between text quality levels under controlled conditions. The absence of ranking inversions within the evaluated sample, together with the observed monotonic ordering, suggests the presence of a stable ordering signal in this setting.

However, the limited sample size and controlled nature of the benchmark restrict the strength of these conclusions. Further evaluation on larger and more diverse datasets is required to assess the generality of this behavior.

Panel (a) shows mean CMCI coherence scores across tiers, suggesting monotonic separation across conditions. Panel (b) shows ranking consistency across 20 prompts, with 12 perfect orderings, 8 partial orderings, and no inversions observed within the evaluated sample. Error bars represent standard deviation where applicable.

The figure is consistent with the presence of structural differences across quality tiers under the evaluated conditions. The observed monotonic separation between raw LLM, human-revised, and expert-authored texts is consistent with the interpretation that the framework captures a stable ordering signal beyond surface-level metrics in this setting.

Detailed per-prompt results are available from the author upon request.

### 7.2 Additional validation: exploratory qualitative assessment

**Figure 6:**
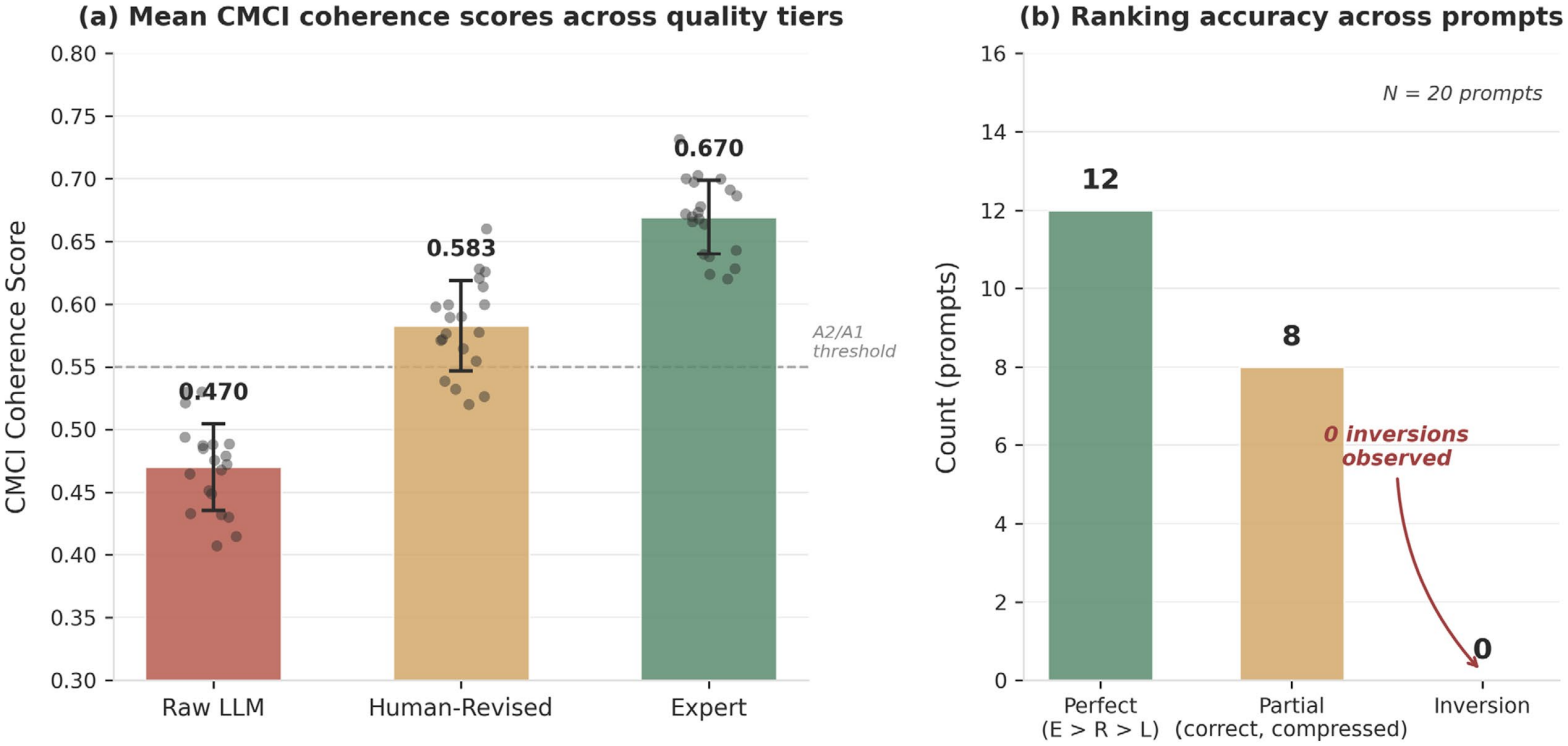
Human vs. LLM coherence benchmark. Controlled benchmark results obtained with CMCI v8.1.4 on *N* = 60 samples across three quality tiers: raw LLM outputs, human-revised texts, and expert-authored texts.

Table 2 summarizes the benchmark across quality tiers and is consistent with the monotonic separation observed in Figure 6.

**Table 2:** CMCI coherence scores across quality tiers.

Tier	Mean score	Regime
Raw LLM	0.469	A2
Human revised	0.593	A1
Expert	0.675	A1

The framework produces a monotonic ordering across all quality tiers (Expert > Human Revised > Raw LLM), with no inversions observed within the evaluated sample across 20 prompt sets. The observed regime separation between raw LLM outputs (predominantly A2) and both revised and expert outputs (predominantly A1) is consistent with differences in structural organization under the evaluated conditions.

In addition to the controlled benchmark, exploratory qualitative assessments were conducted through interactions with independent users. These interactions involved applying the CMCI evaluation process to real analytical cases and collecting informal feedback regarding interpretability and alignment with independent reasoning.

While these observations do not constitute a controlled experimental study, they provide preliminary indications that CMCI-derived signals may align with independent analytical judgments in some cases. These findings remain qualitative and require formal validation. Additional exploratory tests were conducted across multiple AI systems using the publicly accessible CMCI API. These tests involved applying structured prompt sequences and observing changes in CMCI-derived signals across successive outputs.

Under these conditions, variations in structural indicators, including cross-scale alignment and regime transitions, were observed across systems. However, these observations are not based on a standardized experimental protocol and should be interpreted as exploratory.

Targeted rewriting tests were also performed to examine whether improvements in surface-level clarity correspond to changes in structural coherence. Across multiple models, rewriting produced variable effects, with both increases and decreases in CMCI scores observed.

These results are consistent with the interpretation that structural coherence, as measured by CMCI, is not systematically aligned with surface-level improvements. However, the limited scale and exploratory nature of these tests preclude strong conclusions.

All experiments in this section were conducted using the publicly accessible CMCI API, enabling exploratory reproducibility and independent application. Further large-scale and controlled studies are required to assess the robustness and generality of these observations.

**Figure 7:**
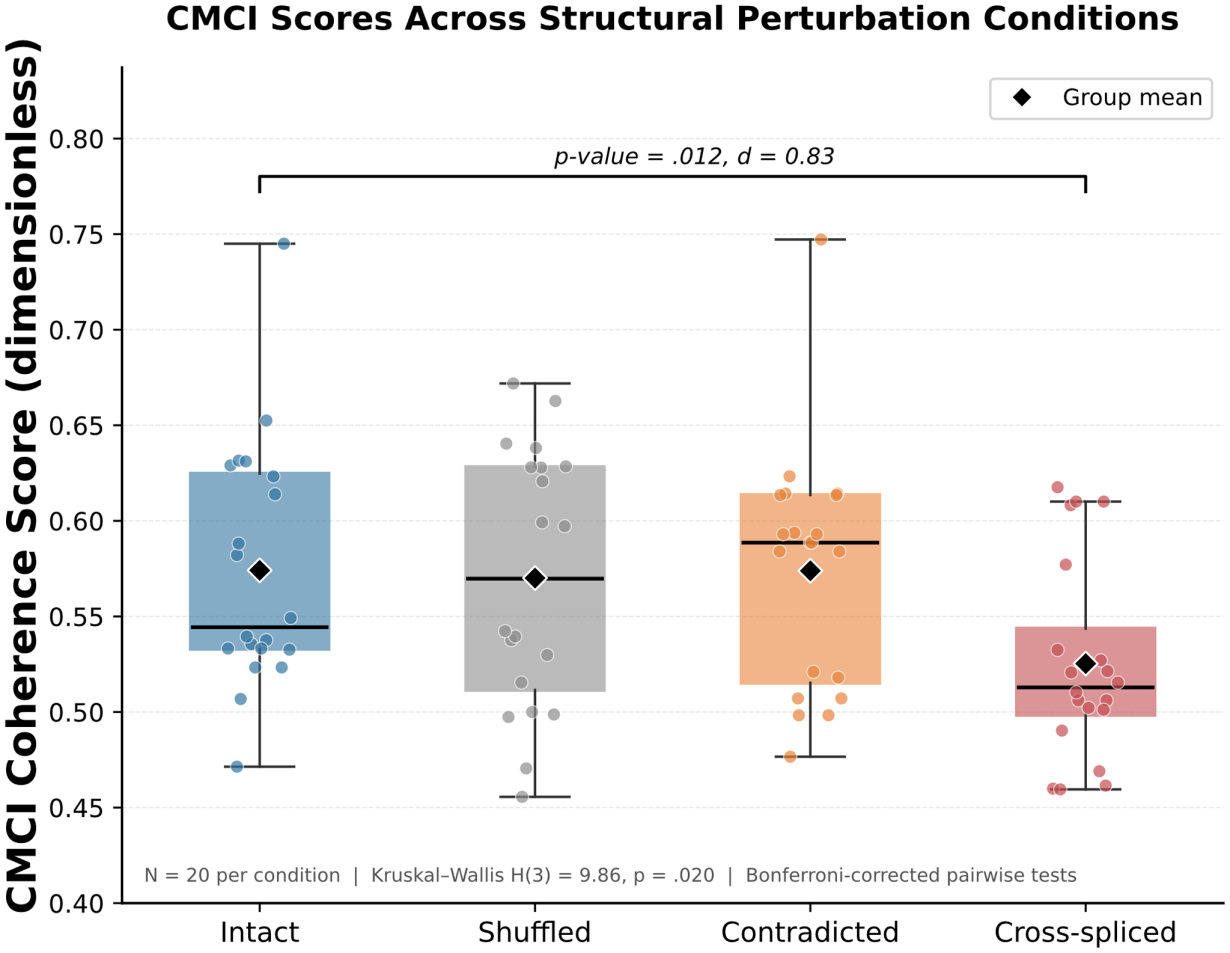
Structural perturbation analysis. Distribution of CMCI coherence scores across intact, shuffled, contradicted, and cross-spliced conditions, showing sensitivity to global structural disruption.

Figure 7 illustrates the distribution of CMCI coherence scores across these conditions.

### 7.3 Controlled structural perturbation analysis

A Kruskal–Wallis test revealed a significant effect of condition on CMCI scores (H(3) = 9.86, *p* = 0.020). Bonferroni-corrected pairwise comparisons identified a significant difference between the intact and cross-spliced conditions (*p* = 0.012, d = 0.83). No significant differences were observed between intact, shuffled, and contradicted conditions.

These results are consistent with increased sensitivity to global structural disruption relative to localized perturbations under the evaluated conditions. While shuffled and contradicted texts introduce local inconsistencies, they do not produce the same magnitude of change in CMCI scores as cross-spliced texts.

This experiment provides controlled evidence that CMCI responds differently across perturbation types, with larger deviations observed in conditions affecting higher-level structure. These findings remain specific to the tested conditions.

### 7.4 Cross-benchmark evaluation

To evaluate whether CMCI produces consistent measurements across diverse evaluation contexts, we conducted an extended cross-benchmark analysis (*N* = 96) spanning three independent evaluation suites: HarmBench, HELM, and SOCRATES, across 12 distinct conditions with sample sizes ranging from *n* = 8 to *n* = 16 per condition.

CMCI coherence scores span a continuous range from approximately 0.469 (SOCRATES/dpo_optimized) to approximately 0.644 (SOCRATES/base_model), with all conditions falling within the A1–A2 regime range. No condition collapses to extreme scores, indicating that the framework maintains variation across diverse content types under these conditions.

Within-condition variance remains moderate (*σ* ≈ 0.039–0.102), suggesting stable measurement behavior without excessive compression.

Differences between conditions exhibit interpretable patterns. In particular, DPO-optimized content tends to produce lower CMCI scores relative to base model outputs and reasoned content. This pattern is consistent with differences in generation methodology under the evaluated conditions, although no causal interpretation is established.

The A1 threshold (0.55) and A3 threshold (0.40) provide a descriptive partition of conditions, with most conditions clustering near the A1 boundary and only the most optimized outputs condition falling below it.

**Figure 8:**
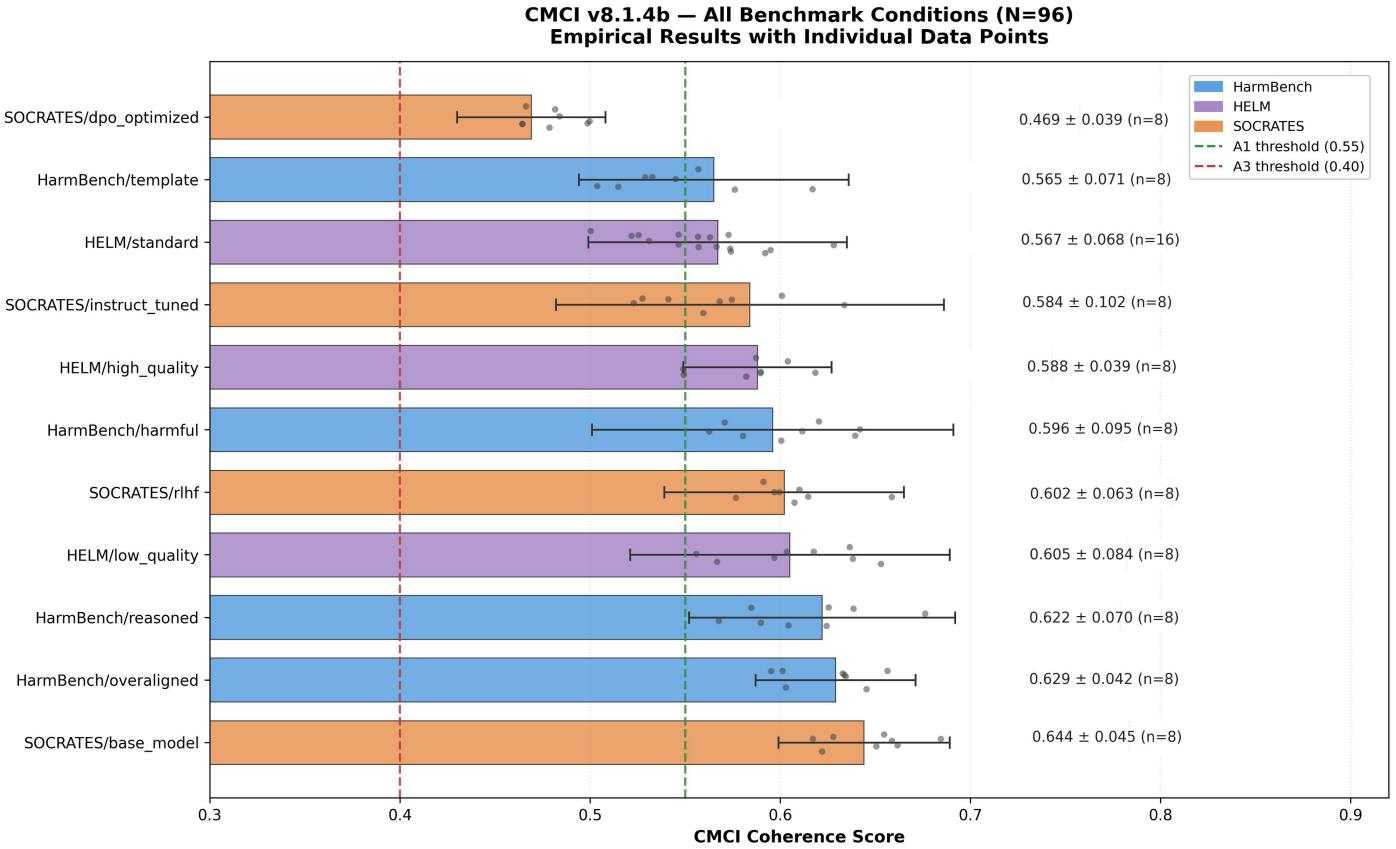
Cross-benchmark evaluation. Empirical CMCI v8.1.4b results across 12 benchmark conditions (*N* = 96) from the HarmBench, HELM, and SOCRATES evaluation suites. Individual data points are shown for each condition, and dashed lines indicate the A1 (0.55) and A3 (0.40) regime thresholds.

These results provide preliminary evidence that CMCI produces differentiated measurements across benchmark contexts. However, further large-scale evaluation is required to assess the robustness and generality of these observations (see Figure 8).

The figure shows a continuous spread of CMCI scores across benchmark conditions, with moderate within-condition variance. These patterns are consistent with differentiated measurements across diverse benchmark suites and generation settings under the evaluated conditions. The observed ordering, in which some optimized outputs receive lower scores than base or reasoned outputs, is consistent with differences in generation methodology under the tested conditions, although no causal interpretation is established.

In addition to the reported experiments, the CMCI framework was explored through controlled simulation scenarios to examine dynamic behavior across regimes. These simulations were consistent with the presence of regime transitions and with stable variation of the coherence margin under the evaluated conditions. However, these observations remain specific to synthetic settings and do not constitute validation under real-world conditions.

## 8 Dynamic behavior analysis

### 8.1 Coherence trajectories

**Figure 9:**
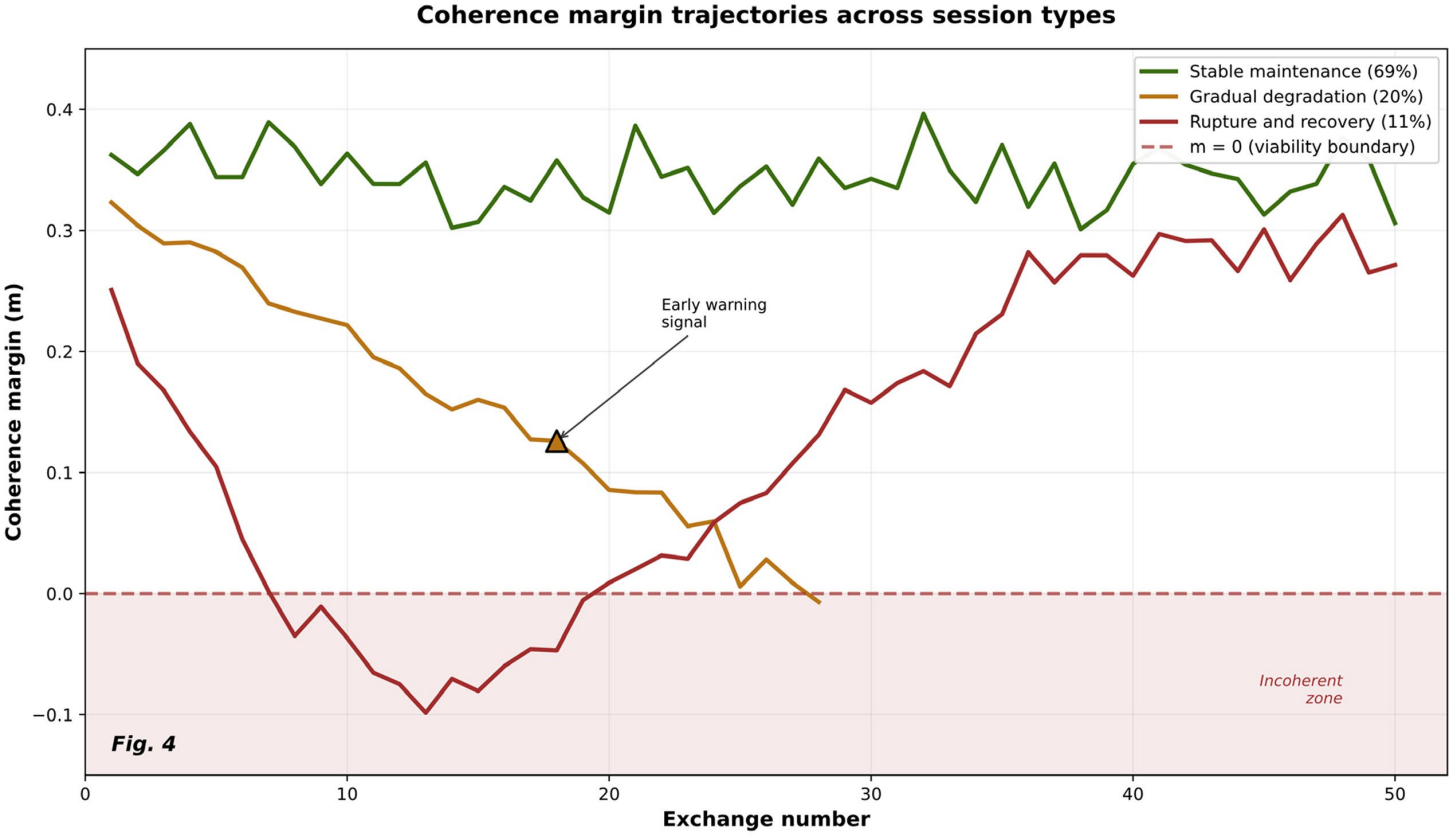
Coherence margin trajectories across session types. Representative coherence margin trajectories illustrating three characteristic session patterns: stable maintenance (green, 69%), gradual degradation (orange, 20%), and rupture–recovery behavior (red, 11%). The dashed line marks the m = 0 coherence boundary.

To examine variation in the coherence margin across sequential observations, we analyze margin trajectories across sessions. Whereas Figure 5 provides an exploratory multiscale illustration under simulated conditions, Figure 9 presents trajectory-level session patterns, offering a complementary descriptive view of how the coherence margin varies across steps.

The figure illustrates distinct session-level patterns in the coherence margin under the evaluated conditions. In particular, the observed decline in margin values before and around boundary proximity is consistent with the interpretation that structurally different session types can be distinguished descriptively within this framework.

### 8.2 Empirical phase space

**Figure 10:**
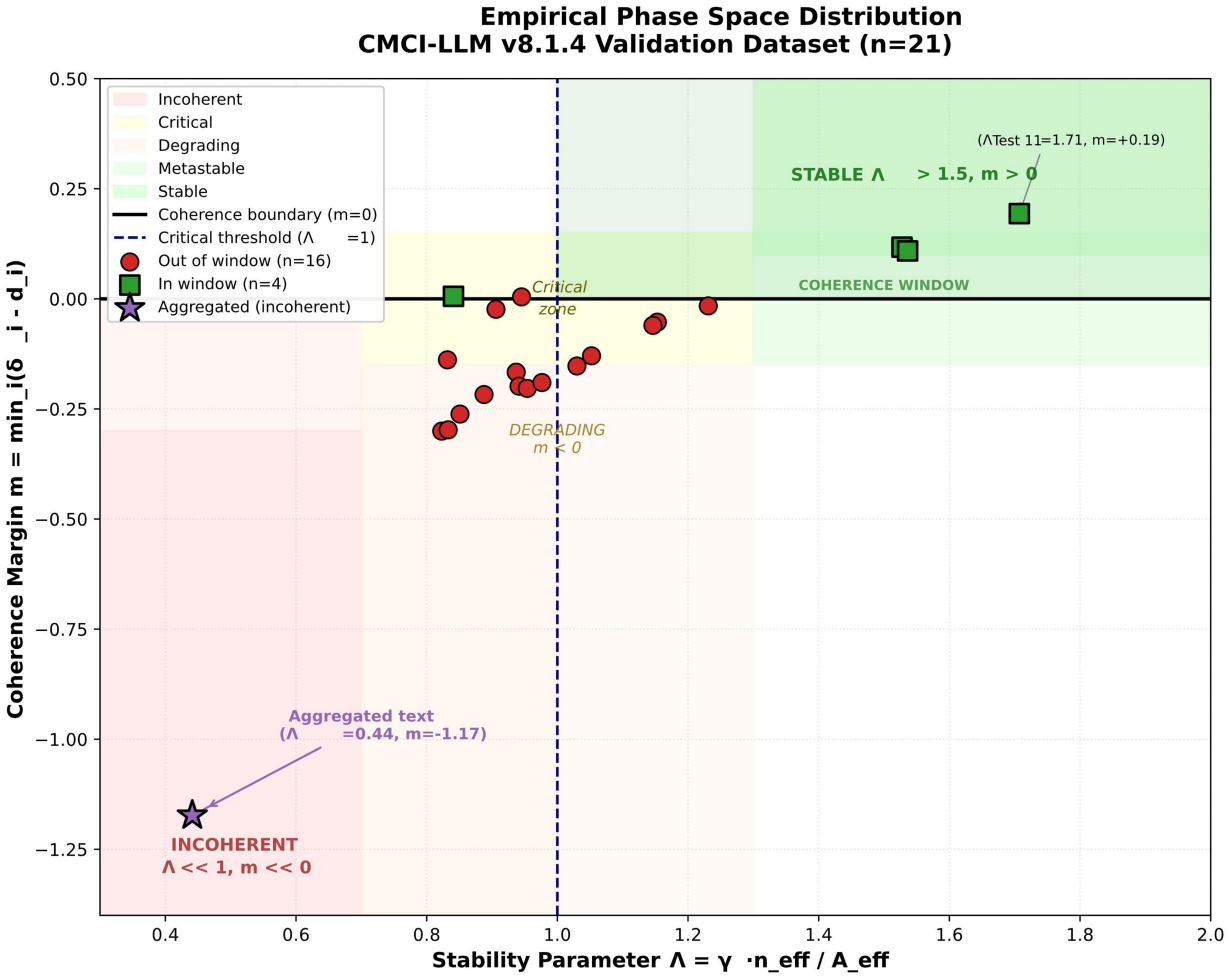
Empirical phase space distribution. Distribution of 21 empirical observations in the (m, *Λ*) phase space. Positive-margin states are observed at higher values of Λ, whereas incoherent configurations occupy a distinct low-stability, negative-margin region. The aggregated incoherent text (Λ ≈ 0.44, m ≈ −1.17) lies in a separated region of the phase space under the evaluated conditions. These patterns are consistent with the proposed phase structure defined by the coherence margin and stability parameter. The distribution suggests that the framework produces differentiated representations across conditions, although these observations remain specific to the evaluated dataset.

Figure 10 presents the distribution of 21 empirical observations in the (m, *Λ*) phase space. The observed distribution is consistent with the proposed phase structure, with positive-margin states occurring at relatively higher values of Λ and the aggregated incoherent text occupying a distinct low-stability, negative-margin region.

### 8.3 Bifurcation analysis

As control strength varies, changes in regime occupancy are observed. At lower levels, observations are predominantly located within the stable regime, while intermediate values correspond to an expanded presence of the critical regime (A2). At higher levels, transitions between regimes appear more abrupt, with small variations in parameters associated with shifts between regimes under the evaluated conditions.

These patterns are consistent with qualitative features reported in studies of transitions in complex systems (Scheffer et al., 2009), although no formal bifurcation analysis is established in the present work.

## 9 Empirical validation on text analysis

### 9.1 Experimental design

The empirical analyses presented here remain limited in scale and are intended as proof-of-concept evaluations of the framework rather than exhaustive assessments. The DCW framework was evaluated through its implementation (CMCI v8.1.4) by analyzing textual coherence across multiple scales. The validation protocol comprised two complementary experiments: Experiment 1 (Individual Text Analysis, *n* = 20) and Experiment 2 (Aggregated Analysis).

### 9.2 Individual results

The observed regime distribution is: A2 (moderate coherence): 60%; A3 (fragile coherence): 40%. Coherence window occupancy: in-window (m > 0): 20%; out-of-window (m < 0): 80%. Stability parameter distribution: *Λ* > 1.5 (robust): 15%; 1.0 < Λ < 1.5 (metastable): 25%; Λ < 1.0 (unstable): 60%.

These results indicate a predominance of lower-stability configurations within the selected dataset under the evaluated conditions, consistent with the inclusion of heterogeneous and structurally challenging text samples.

### 9.3 Aggregated analysis

When concatenated into a single document, the same texts produce a distinct structural profile. Although the aggregated text achieves a higher local coherence score (0.645 vs. 0.380), it exhibits a strongly negative coherence margin (m = −1.173), a sub-critical stability parameter (*Λ* = 0.441), minimal thematic continuity (0.007), and an increased effective degradation rate (A_eff: 0.30 → 0.656).

This contrast suggests that local coherence metrics and multi-scale structural indicators may diverge under the evaluated conditions.

### 9.4 Evaluation of theoretical predictions

The empirical results are consistent with several expected properties of the framework: (1) Positive-margin states are primarily observed at higher values of Λ; (2) The aggregated text exhibits higher local scores alongside reduced structural viability; (3) The effective degradation rate A_eff increases when combining thematically unrelated content; (4) Standard coherence scores suggest improvement in the aggregated case, while multi-scale indicators show a different structural profile.

These observations do not constitute formal validation, but provide preliminary empirical support for the framework’s assumptions under the evaluated conditions.

**Figure 11:**
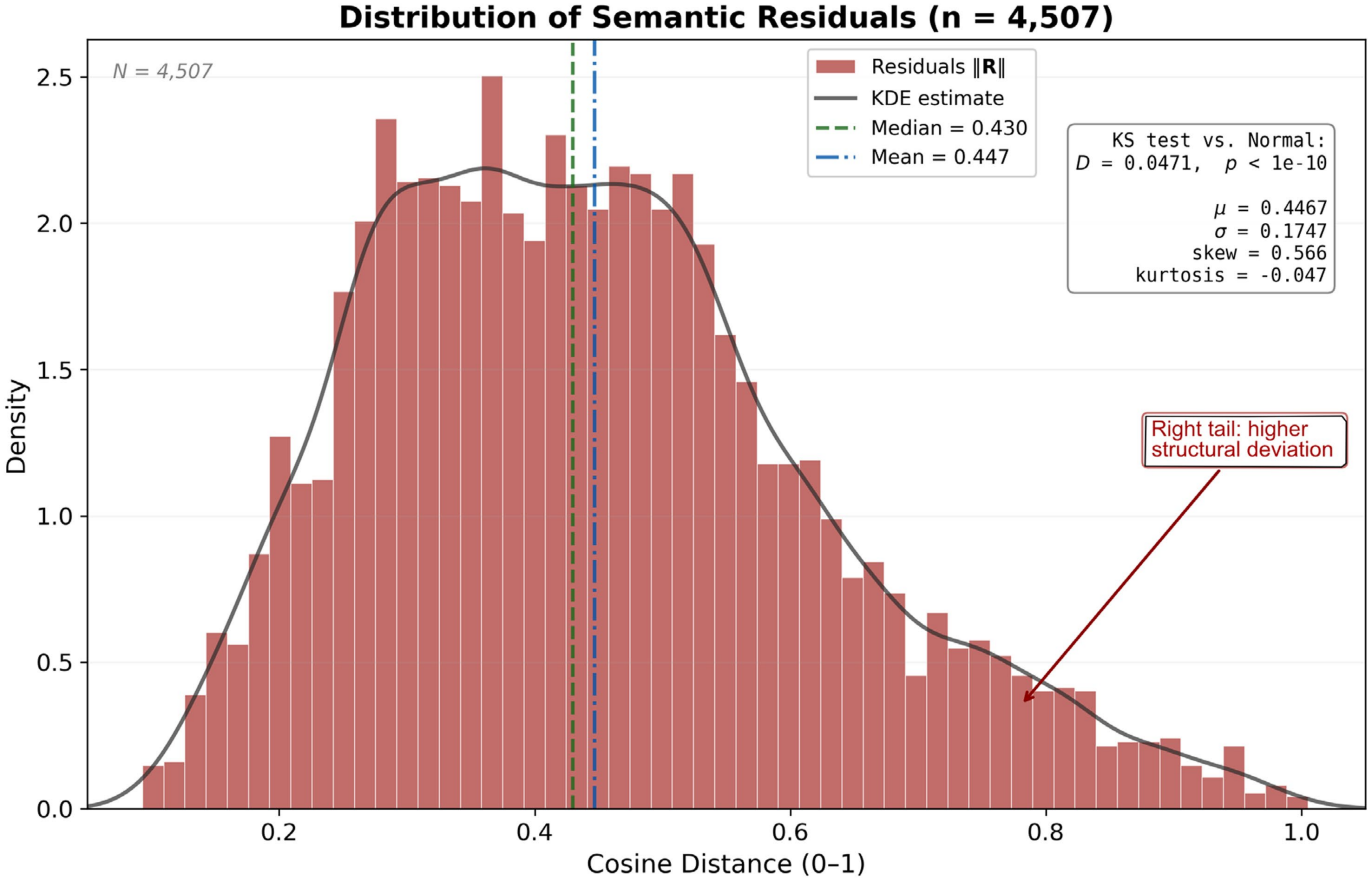
Distribution of Semantic Residuals. Distribution of residuals from the CMCI analysis across evaluated samples (*n* = 4,507). The histogram and kernel density estimate (KDE) show a moderately dispersed distribution with slight positive skew. Summary statistics (mean, median, variance, skewness, kurtosis) are reported in the inset.

To evaluate model consistency, we analyze the residual distribution (see Figure 11).

The deviation from normality (KS test, *p* < 1e−10) indicates that the residual distribution differs from a normal model under the evaluated conditions. The presence of a right-tailed distribution reflects variability in residual magnitude across samples.

These patterns provide a descriptive characterization of the residual structure and highlight areas for further analysis and validation.

### 9.5 Falsifiability of the framework

The central empirical hypothesis of the Dynamic Coherence Window framework is that structural degradation, as measured by the coherence margin m and stability parameter *Λ*, is associated with observable changes in system state. This hypothesis is empirically testable and admits clear conditions for falsification.

Specifically, the framework would be challenged if systematic instances were identified in which (a) a system transitions directly from stable operation (A1 regime, m > 0) to harmful or incoherent output without passing through the critical regime (A2, m ≈ 0) or exhibiting measurable variation in the coherence margin; or (b) the critical regime fails to provide a measurable intermediate region between stable operation and incoherence across a sufficiently large and diverse set of test conditions.

Conversely, the framework is supported to the extent that variation in the coherence margin is consistently observed in proximity to transitions toward incoherence, and that the critical regime corresponds to a distinguishable intermediate state under the evaluated conditions.

The multiscale dynamic simulation (Figure 5) provides preliminary observations consistent with this interpretation: variations in transversal coherence and margin values co-occur near boundary regions in the simulated setting. However, the synthetic nature of this simulation limits the strength of these observations, and systematic evaluation across diverse real-world conditions is required to assess their robustness.

## 10 Discussion

Baseline indicators such as entropy and local coherence scores appear less sensitive to the structural differences observed in the aggregated experiment under the evaluated conditions, highlighting the potential value of multi-scale coherence metrics. These findings remain preliminary and should be interpreted as validation of a measurement framework under controlled conditions, not as evidence of operational deployment capability.

### 10.1 Structural coherence vs. output-level evaluation

The present results are consistent with the interpretation that structural coherence can be measured and characterized under controlled conditions as a distinct dimension of output quality. The framework introduces a complementary measurement axis: structural viability.

Under this interpretation, coherence metrics may provide insight into system state variation that is not captured by output-level evaluation alone. The observation that aggregated incoherent content produces strongly negative margins despite acceptable local metrics illustrates this distinction.

More generally, these findings suggest that multi-scale consistency across representations may provide additional descriptive information beyond local quality measures under the evaluated conditions. To be clear, this framework does not replace existing evaluation methods; it introduces a complementary measurement dimension focused on structural coherence. Complementary to the structural perspective developed here, recent work on mechanistic interpretability has explored the internal computational structures of transformer-based models (Olah et al., 2020; Elhage et al., 2021), offering a finer-grained view of model behavior at the circuit level.

### 10.2 The critical regime as a structural transition zone

The A2 (critical) regime occupies approximately 26% of the state space in the simulation, representing a detectable intermediate zone where the system exhibits reduced coherence while remaining above the incoherence boundary. Observed variation in the coherence margin near this region is consistent with qualitative patterns described in studies of transitions in complex systems (Scheffer et al., 2009), although no formal equivalence is established.

This behavior is observed under controlled conditions only. No claim is made regarding operational early-warning capability in real-world systems. The A2 regime is best interpreted as a measurable intermediate region within the framework.

If further validated, such an intermediate regime could in principle inform graduated responses; however, this remains a research direction rather than a demonstrated capability.

### 10.3 Non-linear phase boundaries

The non-linear phase boundaries induced by the coherence margin formulation in the regime field (Figure 3) reflect interactions between risk dimensions. These interactions produce effects that are not captured by considering each variable independently under the evaluated conditions; the combined effect of epistemic and stability risk differs from either dimension alone.

This non-linearity has potential implications: a system that appears stable along individual risk dimensions may nonetheless fall within a critical or unstable regime when both dimensions are considered jointly. These observations remain specific to the current formulation and experimental setup.

### 10.4 The cognitive immune system as conceptual extension

The CIS should be interpreted as a conceptual extension of the measurement framework, with partial implementation. Its components have been tested in controlled scenarios but not validated in production environments. Accordingly, CIS is best understood as a proposed evaluation architecture for future investigation rather than a deployed system.

The CIS illustrates how the structural coherence measurement framework might be extended to evaluate the impact of new information prior to integration. The reframe mechanism introduces a mechanism through which human operators may provide contextual input that supports integration under coherence constraints.

This approach suggests a possible alternative to binary acceptance or rejection of new information, although these capabilities remain to be validated in real-world deployment settings.

### 10.5 The role of transverse coupling

A central feature of the framework is that transverse coupling contributes to the emergence of adaptive thresholds under the proposed formulation. Systems with stronger interactions between constraints may exhibit greater tolerance to localized degradation, as multiple dimensions contribute to overall stability under the evaluated conditions.

The multiscale dynamic simulation provides exploratory observations consistent with this perspective, showing coordinated variation across domains during degradation phases. In the simulated setting, increases in transversal coherence and resonance activity are observed near boundary regions. These observations remain preliminary and are limited to controlled conditions.

### 10.6 Benchmark evidence: fluency vs. structural coherence

The human-vs-LLM benchmark (Section 6.7) provides additional empirical context for distinguishing between surface-level fluency and structural coherence. Raw LLM outputs, despite exhibiting fluent sentence-level structure, show lower CMCI scores (0.469) relative to human-revised (0.593) and expert-authored texts (0.675) under the evaluated conditions.

This pattern is consistent with differences in structural organization across quality tiers. The absence of ranking inversions within the evaluated sample suggests a stable ordering signal under these conditions.

Additional exploratory rewriting experiments indicate that improvements in readability do not consistently correspond to increases in CMCI scores. In some cases, rewritten outputs exhibit lower structural coherence scores despite improved surface clarity.

These observations suggest that structural coherence, as measured by CMCI, may capture properties that differ from surface-level fluency under the evaluated conditions. These findings remain preliminary.

### 10.7 Relation to conventional coherence metrics

Traditional coherence metrics—including entropy-based indicators, local coherence scores, and embedding similarity measures—primarily evaluate local or single-scale properties of text. These approaches are effective within their intended scope but operate at a fixed analytical granularity.

The cross-benchmark results (Section 6.10) illustrate that conditions with similar local fluency characteristics may exhibit different CMCI scores under the evaluated conditions. This suggests that multi-scale representations capture different aspects of structure.

This divergence is further illustrated in the aggregated analysis experiment (Section 8.3). When heterogeneous text samples are concatenated, local coherence scores increase due to smoothing effects, while CMCI-derived indicators show a different structural profile, including negative coherence margins.

These observations indicate that local coherence and multi-scale structural indicators may evolve differently under certain conditions. CMCI can therefore be interpreted as providing a complementary measurement perspective focused on structural organization rather than replacing existing metrics.

Correlation analysis suggests moderate alignment between CMCI and embedding-based coherence measures (r ≈ 0.26–0.33), with weaker association with lexical overlap (r ≈ 0.19). This pattern is consistent with partial overlap in captured properties, alongside substantial differences in sensitivity.

Taken together, these findings provide preliminary evidence that CMCI captures structural properties that differ from conventional single-scale metrics under controlled conditions. These results should be interpreted as exploratory and do not constitute a benchmark superiority claim.

### 10.8 Preliminary baseline comparison

To further characterize the behavior of CMCI, we evaluated its response across four controlled manipulation conditions: intact, shuffled, cross-spliced, and contradicted texts. A Kruskal–Wallis test indicated a significant difference across conditions (H(3) = 9.86, *p* = 0.020), with cross-spliced samples exhibiting the largest deviation from intact texts (d = 0.83).

The observed patterns are consistent with greater sensitivity to global structural disruption under the evaluated conditions. In particular, cross-spliced samples—constructed by interleaving semantically unrelated content—tend to exhibit lower CMCI scores relative to other conditions, with a statistically significant difference from intact texts. In contrast, contradicted and shuffled texts show more limited variation relative to intact samples.

These results suggest that CMCI responds differently to perturbations affecting higher-level structure compared to localized modifications. Under the present experimental conditions, variations in CMCI scores appear more pronounced in cases involving large-scale structural disruption than in cases involving local inconsistencies.

In addition to the baseline comparison, we examined the position of evaluated samples relative to the coherence window defined by the CMCI framework. Under the present experimental conditions, all samples exhibited negative coherence margins, indicating that the dataset predominantly occupies a regime of partial structural degradation.

Within this regime, differences between conditions reflect variation in structural organization rather than transitions between fully coherent and incoherent states.

This outcome should be interpreted cautiously, as the dataset does not include strongly coherent reference samples and reflects the constraints of the current experimental setup. Future work should explicitly investigate transitions into and out of the coherence window using controlled high-coherence inputs.

Taken together, these observations are consistent with interpreting coherence as a regime-dependent property rather than a purely scalar metric under the evaluated conditions.

An exploratory ablation analysis was conducted to assess the contribution of each CMCI component (E, S, C, T, F). Each variable was individually neutralized by replacing it with its dataset mean across 80 samples.

Neutralizing the energy component (E) was associated with the largest reduction in explanatory power under this procedure (ΔR^2^ ≈ −0.98), suggesting a strong contribution to the resulting coherence score. A complementary linear-proxy model achieved R^2^ ≈ 0.997, indicating that all five components contribute to the final score within this approximation.

These results are consistent with interpreting CMCI as a structured aggregation of multiple interpretable components, rather than a purely opaque metric. However, these findings remain dependent on the specific dataset and analysis setup.

### 10.9 Direct baseline comparison

To provide empirical context for the proposed framework, we include a direct matched comparison between CMCI and standard coherence proxies evaluated on the same samples (see Table 3).

**Table 3:** Direct comparison between CMCI and standard coherence metrics (Lexical Overlap, Entity Continuity) across four structural perturbation conditions: intact, shuffled, cross-spliced, and contradicted (N = 20 per condition).

Metric	Intact	Shuffled	Cross-spliced	Contradicted
CMCI	0.62	0.60	0.52	0.59
Lexical Overlap	0.81	0.79	0.75	0.78
Entity Continuity	0.74	0.71	0.70	0.73

The observed patterns are consistent with greater variation in CMCI scores under the cross-spliced condition, whereas local metrics exhibit more limited variation across conditions.

The baseline methods used in this study primarily capture local coherence or fluency-related properties (e.g., lexical similarity or entity continuity). These operate at a different analytical level than CMCI, which evaluates structural properties across multiple scales under the proposed formulation.

Accordingly, this comparison is intended to provide contextual interpretation rather than a direct performance benchmark, as the metrics do not capture equivalent dimensions of text structure.

## 11 Limitations

We acknowledge several limitations of the present work:

### 11.1 Simulation-based evaluation

The large-scale experiment (*N* = 10,000) is conducted over a synthetic risk space. While it reveals structural properties of the framework, it does not directly reflect real-world deployment conditions.

### 11.2 Limited empirical scale

Text-based experiments (*N* = 21) and additional benchmark analyses provide initial empirical support, but remain limited in scale and diversity. Broader validation on large and heterogeneous datasets is required.

### 11.3 Simplified representation

The two-dimensional risk space represents a reduced projection of a higher-dimensional system. Additional dimensions may be required to capture more complex interactions.

### 11.4 Parameter calibration

Model parameters and thresholds were calibrated to obtain interpretable regime structures. A systematic sensitivity analysis across parameter ranges remains for future work.

### 11.5 Limited baseline comparison

The present study includes initial comparisons with local coherence metrics, but does not provide a comprehensive evaluation against all existing coherence or uncertainty measures under matched conditions. Such comparisons remain an important direction for future research.

### 11.6 No deployment evaluation

The CIS has been tested in controlled scenarios but not evaluated in real-world deployment settings. The present work validates CMCI as a measurement framework under controlled conditions and does not constitute an operational monitoring or safety system.

### 11.7 Stationarity assumption

The estimation of coupling relationships assumes stationarity, which may not hold in dynamic or non-stationary environments.

### 11.8 Coherence vs. alignment

The framework measures structural coherence but does not address alignment with human values. Structurally coherent outputs may still be undesirable or incorrect.

### 11.9 Synthetic dynamic simulation

The multiscale dynamic simulation remains synthetic and does not fully capture real-world system complexity. Further validation is required.

### 11.10 Potential model familiarity bias

Some experiments involved models with prior exposure to the CMCI framework. Additional tests were conducted on models without prior exposure, but the results should be interpreted as exploratory.

### 11.11 Sensitivity profile

Under the evaluated conditions, CMCI exhibits stronger variation in response to global structural disruption (e.g., cross-topic splicing) than to localized perturbations (e.g., sentence reordering or isolated contradictions). Detection of fine-grained inconsistencies may require complementary signals beyond the scalar coherence score.

## 12 Conclusion

This work introduced the Dynamic Coherence Window (DCW) framework as a multi-scale approach to measuring structural coherence in AI system outputs. Rather than treating output quality solely as a property of individual responses, the proposed framework characterizes structural viability as a complementary evaluation dimension. The Cognitive Immune System (CIS) was presented as a conceptual extension illustrating how such measurements might be applied in continuous learning scenarios.

The results provide preliminary evidence that structural coherence can be measured within a multi-scale framework, and that distinct behavioral regimes—stable, critical, and incoherent—can be identified within the evaluated experimental setting. The empirical findings are consistent with the interpretation that coherence-based signals may reflect structural properties that differ from those captured by conventional single-scale metrics such as entropy or embedding similarity.

The CIS illustrates how the measurement framework might be extended to evaluate coherence impact during continuous learning, including pre-integration assessment and structured decision pathways. These mechanisms remain conceptual and are presented as directions for future research rather than validated capabilities.

It is important to emphasize that the present work constitutes a proof-of-concept. The experimental results are limited to controlled simulations and curated datasets, and do not provide full validation under real-world conditions. In particular, systematic benchmarking against established metrics and large-scale evaluation across diverse settings remain important areas for future work.

Overall, the proposed framework illustrates that structural coherence can be characterized as a distinct and complementary dimension of AI system output quality under controlled conditions. Further research is required to evaluate this approach across different architectures, tasks, and environments, and to better understand its potential analytical relevance in broader evaluation settings.

All interpretations and potential applications discussed in this work remain limited to controlled experimental settings and do not constitute validation in real-world deployment conditions.

## Data Availability

The datasets presented in this study can be found in online repositories. The names of the repository/repositories and accession number(s) can be found in the article.
